# Pan-Cancer Prediction of Genomic Alterations from H&E Whole-Slide Images in a Real-World Clinical Cohort

**DOI:** 10.3390/genes17040371

**Published:** 2026-03-25

**Authors:** Dongheng Ma, Hinano Nishikubo, Tomoya Sano, Masakazu Yashiro

**Affiliations:** 1 Department of Molecular Oncology and Therapeutics, Osaka Metropolitan University Graduate School of Medicine, 1-4-3 Asahimachi, Abeno-ku, Osaka 545-8585, Japan; 2 Cancer Center for Translational Research, Osaka Metropolitan University Graduate School of Medicine, 1-4-3 Asahimachi, Abeno-ku, Osaka 545-8585, Japan

**Keywords:** genomic alteration prediction, computational pathology, whole-slide imaging, pathology foundation model

## Abstract

**Background**: Predicting genomic alterations from routine hematoxylin and eosin (H&E) whole-slide images (WSIs) may help triage molecular testing. **Methods**: We retrospectively enrolled 437 patients at Osaka Metropolitan University Hospital across 26 cancers, matched with clinical gene-panel data. We curated 1023 binary endpoints across SNV, CNV, and SV categories. We extracted slide embeddings from five pathology foundation models (Prism, GigaPath, Feather, Chief, and Titan) using a unified feature extraction pipeline and benchmarked them using a lightweight downstream Multi-Layer Perceptron (MLP) classifier. Using the best-performing patch feature system, we trained a multi-instance learning model to assess incremental benefit. **Results**: Titan achieved the highest and most stable transfer performance, with a median endpoint-wise Area Under the Receiver Operating Characteristic curve (AUROC) of 0.77 in the slide benchmarking; at the patch-level, prediction of *APC*_SNV reached an AUROC of 0.916, and prediction of *KRAS*_SNV reached an AUROC of 0.811 on the held-out test set. **Conclusions**: In a heterogeneous clinical gene-panel setting, pathology foundation models can provide strong baseline genomic-prediction signals without additional fine-tuning. We propose a practical, deployment-oriented two-stage workflow: rapid slide-embedding screening to prioritize robust representations and candidate endpoints, followed by patch-level training for high-value tasks where additional performance gains and interpretable regions are clinically worthwhile.

## 1. Introduction

Genomic profiling is now a cornerstone of precision oncology, informing diagnosis, prognostic stratification, and therapy decisions [1]. Multigene-targeted sequencing panels are increasingly adopted because they provide a practical balance between breadth, turnaround time, and clinical actionability, supported by the expanding landscape of biomarker-linked drug indications and companion diagnostics [2]. However, routine implementation still faces real-world friction like limited tissue availability, as well as cost and turnaround-time constraints, which motivate complementary strategies that can triage and prioritize genomic testing. In practice, timely molecular profiling is not always feasible, as sequencing often requires sufficient tumor tissue of adequate quality, and can take 4–6 weeks to generate results, potentially delaying treatment decisions in rapidly progressing cancers. These challenges are particularly pronounced in patients with small biopsies, degraded archival specimens, or in resource-limited settings where comprehensive panel testing is not consistently available. In this context, recent advances in computational pathology suggest leveraging routine H&E whole-slide images to infer molecular alterations and provide actionable pre-test cues for downstream testing pathways [3].

Pathology is the gold standard for tumor diagnosis, and beyond conventional diagnostic elements, it captures a spectrum of phenotypic features that are linked to underlying genomic alterations [4]. Several studies have reported associations between specific driver events and interpretable histologic patterns; for example, Liu et al. [5] suggested that the *KRAS* G12D point mutation may relate to tumor immune microenvironment states, and Fanaroffa et al. [6] reported correlations between gene alterations (e.g., *TP53*) and histologic features in pleural mesothelioma. Taken together, these observations support a biological link whereby driver mutations shape cellular architecture, nuclear morphology, stromal composition, and immune contexture, thereby providing a mechanistic basis for the computational prediction of genomic alterations from H&E images. Beyond these human-interpretable correlates, deep learning has made substantial progress in extracting subtle morphologic signals from pathology images to predict genomic alterations, providing a scalable framework to connect tissue morphology with molecular endpoints [7,8,9].

Molecular prediction from H&E whole-slide images has co-evolved with advances in computational pathology. Early studies largely relied on convolutional neural network (CNN)-based tile representation learning and established that routine histomorphology contains computable signals linked to driver genomic events; for example, Valieris et al. [10] reported mismatch repair deficiency prediction in gastric cancer with an area under the receiver operating characteristic curve (AUROC) of 0.81. Subsequently, weakly supervised multiple instance learning (MIL) made end-to-end whole-slide images (WSI) modeling feasible using slide-level labels, enabling broader linkage to molecular endpoints [11]. Cui et al. [12] applied a CNN-based MIL framework to predict *IDH1* mutation in glioma tissue, achieving an AUROC of 0.84. With the rise of large-scale self-supervised learning (SSL) in 2023, SSL-pretrained encoders combined with attention-MIL gained traction for label-efficient and more transferable representations; Zheng et al. [13] reported tumor mutational burden(TMB) prediction in clear cell renal cell carcinoma with external validation AUROC of 0.83. From 2024 onward, foundation models pretrained on massive, diverse WSI collections further improved baseline performance and generalizability. Xu et al. [14] proposed Prov-GigaPath, pretrained on 171,189 WSIs from more than 30,000 patients, and reported performance for five common gene mutation prediction tasks in lung adenocarcinoma, with an average macro-AUROC of 0.626.

Despite these advances, real-world clinical deployment must address mixed tumor types, highly sparse endpoints, and distribution shifts that are common in routine gene-panel cohorts [15]. Whether pathology foundation models remain robust and practically useful in this complex setting remains an open question. Existing studies have predominantly relied on public datasets such as TCGA, with limited cancer type diversity and endpoint coverage. Few studies have compared multiple foundations in pan-cancer in real-world deployment. We hypothesized that foundation model embeddings capture transferable morphologic signals that are informative for genomic alteration prediction, even without fine-tuning, and that a two-stage workflow consisting of rapid slide-level screening, followed by selective patch-level training, could provide a practical framework for molecular test triage and prioritization. Here, we evaluate five foundation models in a local pan-cancer gene-panel cohort of 437 cases. Without fine-tuning, we benchmark slide-level foundation-model representations using lightweight downstream MLP classifiers. Using the best-performing feature system, we further train a patch-level MIL model to assess performance gains, thereby providing a practical view of real-world readiness.

## 2. Materials and Methods

### 2.1. Data Processing

We retrospectively enrolled an initial cohort of 533 patients treated at Osaka Metropolitan University Hospital, Osaka, Japan, between November 2019 and August 2025. These patients had undergone clinical gene-panel testing and had corresponding Hematoxylin and Eosin (H&E)-stained whole-slide images (WSIs) available. The cohort spanned 26 solid tumor types. All slides were scanned using an Aperio CS2 scanner (Leica Biosystems, Nussloch, Germany) at 40× magnification (0.25 μm/pixel). The inclusion criteria were: (i) interpretable gene-panel results; (ii) availability of H&E WSIs from matched surgical or biopsy specimens; and (iii) complete key clinical metadata, including tumor type, sampling site, and date. We applied the following exclusion criteria to refine the dataset: (i) hematological malignancies; (ii) low-quality or severely damaged WSIs (e.g., staining failure, insufficient tissue, or scanning artifacts); (iii) unreliable or non-alignable panel results; and (iv) duplicate cases, for which only the most recent record was retained. After excluding cases based on quality control and tumor type suitability, a total of 437 patients were included in the final analysis. The study protocol was approved by the institutional ethics committee (Approval Nos. 2022-111, 0924, and 2022-077).

### 2.2. Endpoint Definition

Genomic labels were derived from clinical testing platforms (FoundationOne (Foundation Medicine, Inc., Boston, MA, USA), Guardant360 (Guardant Health, Inc., Palo Alto, CA, USA), and GeneMineTop (Konica Minolta REALM, Inc., Tokyo, Japan)), covering approximately 70–700 genes depending on the panel. We grouped alterations into three endpoint categories: 1. SNV: single-nucleotide variants and short insertions/deletions (collectively referred to as “SNV” for simplicity); 2. CNV: copy-number amplification or loss/deletion; and 3. SV: structural variants (e.g., fusions, rearrangements, and other chromosomal structural events). For machine-learning modeling, each “gene × event-type” pair was treated as a binary endpoint (e.g., *TP53*_SNV, *CDKN2A*_CNV, and *ALK*_SV).

### 2.3. Slide Preprocessing

WSI preprocessing and patch extraction were performed using the Trident framework. To ensure compatibility with the downstream feature extractors, patch dimensions were configured to align with the specific input resolution requirements of each foundation model employed. The preprocessing pipeline included automated tissue segmentation to exclude background areas. A rigorous quality control (QC) procedure was then applied to filter out low-quality patches based on the following criteria: presence of significant artifacts such as blur, folds, or pen marks. Finally, we performed stain normalization on all selected patches.

### 2.4. Feature Extraction

We extracted features using the Trident framework and benchmarked five pathology foundation models (Prism [16], GigaPath [14], Feather [17], Chief [18], and Titan [19]) for transfer performance in the local cohort. The architecture, pretraining data scale, and input resolution of each model are summarized in Table 1.

**Figure 1:**
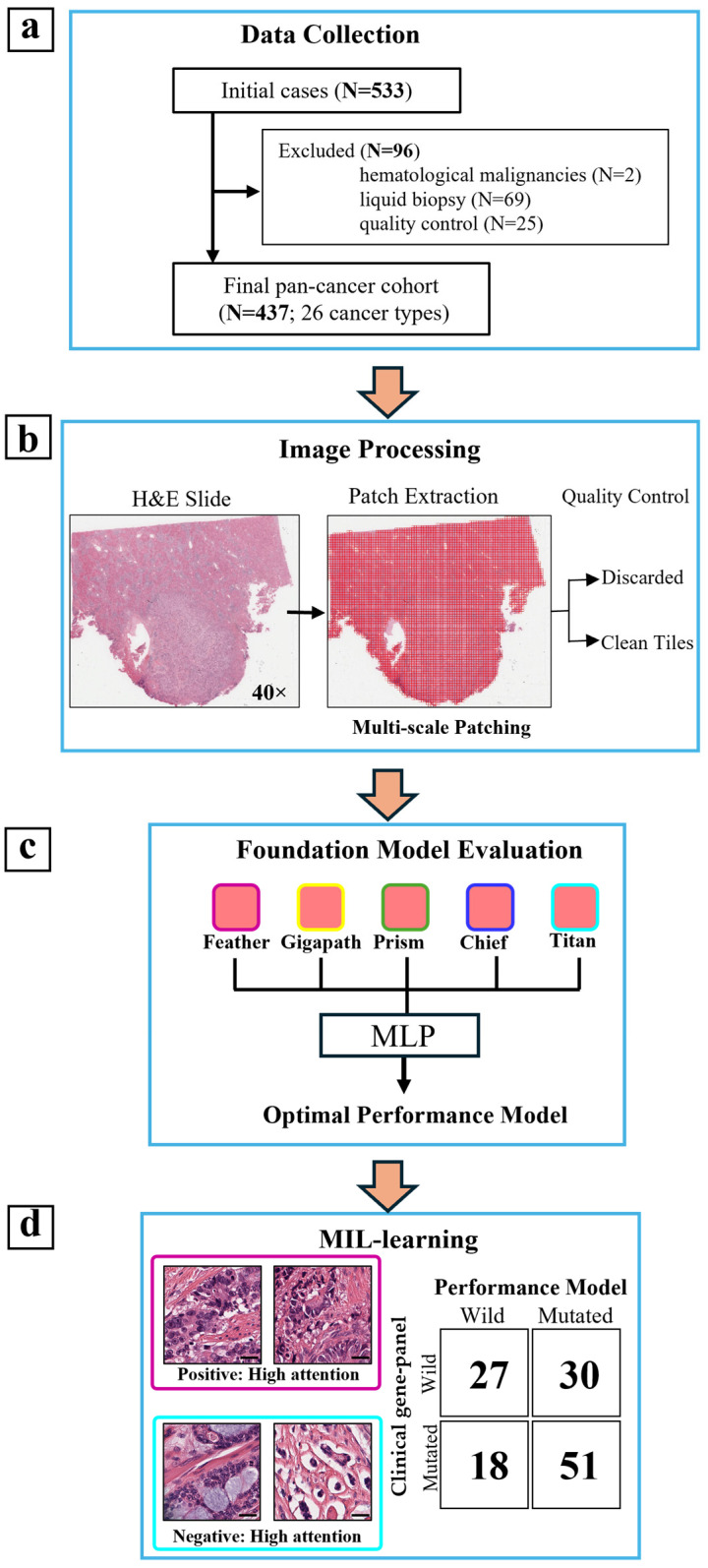
Overview of the study workflow for pan-cancer WSI feature extraction and foundation-model benchmarking. (**a**) Data collection and cohort construction based on clinical gene-panel results, yielding a pan-cancer cohort (N = 437; 26 cancer types). (**b**) Image processing pipeline including H&E slide scanning, patch extraction, multi-scale patching, and quality control to remove low-quality tiles. (**c**) Slide-level features were extracted using the Trident framework from five pathology foundation models (Feather, GigaPath, Prism, Chief, and Titan) and evaluated via a unified downstream predictor MLP to identify the best-performing representation. (**d**) Downstream model evaluation was performed using standard classification metrics (e.g., AUROC curve and confusion matrix) for biomarker prediction. Abbreviations: WSI, whole-slide image; AUROC, the area under the receiver operating characteristic curve; MLP, multi-layer perceptron. Scale bars, 50 µm.

For downstream model training and benchmarking, endpoints were ranked by positive-case prevalence across the full cohort. The top 8 endpoints by positive rate were selected for benchmarking, with the lowest positive rate among selected endpoints being 9.3%. For each model, slide-level features were extracted and utilized for downstream analysis and biomarker prediction. The overall feature extraction and benchmarking workflow is illustrated in Figure 1.

### 2.5. Evaluation

The downstream classifier was implemented as a MLP with two fully connected layers (input → 512 → 1), batch normalization, ReLU activation, and a dropout rate of 0.3. Training was performed using the AdamW optimizer (learning rate = 3 × 10^−4^, weight decay = 1 × 10^−4^) with a batch size of 64 for a maximum of 40 epochs. Class imbalance was addressed by automatic inverse-frequency weighting of the positive class in the binary cross-entropy loss. Early stopping was applied based on validation PR-AUC with a patience of 8 epochs. Input features were standardized using a StandardScaler fitted on the training fold. Model performance was estimated using 3-fold stratified cross-validation, and the mean AUROC across folds is reported.

The primary metric was the area under the receiver operating characteristic curve (AUROC). We additionally reported accuracy, precision, recall, and F1-score. The formula is expressed as

$$AUROC=\int_{0}^{1}TPR\left(FPR\right),dFPR$$

True positive rate (TPR), also referred to as sensitivity or recall, describes the proportion of positive samples that are correctly identified as positive. False positive rate (FPR) describes the proportion of negative samples that are incorrectly predicted as positive, and equals one minus specificity, where specificity is the proportion of negatives correctly predicted as negative.

## 3. Results

### 3.1. Cohort Overview

**Figure 2:**
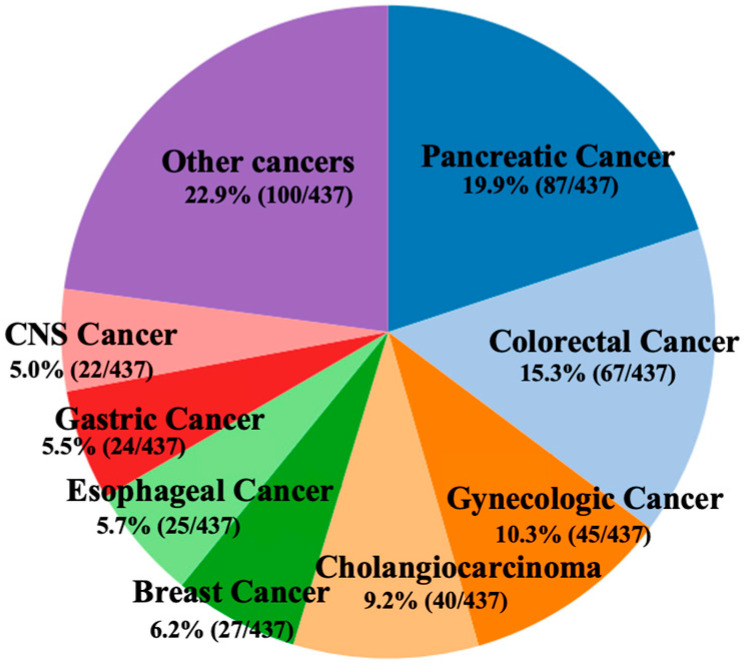
Cancer type distribution. The pie chart summarizes the composition of the final cohort after quality control (N = 437; 26 cancer types). The most frequent tumor categories included pancreatic (n = 87), colorectal (n = 67), gynecologic (n = 45), cholangiocarcinoma/biliary tract (n = 40), breast (n = 27), esophageal (n = 25), gastric (n = 24), and central nervous system (CNS) tumors (n = 22). The remaining cancer types were grouped as “Other” (n = 100).

A total of 533 pathology cases were initially collected. After exclusions due to sampling failure and QC filtering, 437 cases were included in the final analysis, covering 26 cancer types. The most frequent tumor categories were pancreatic cancer (n = 87), colorectal cancer (n = 67), gynecologic cancers (n = 45), cholangiocarcinoma/biliary tract cancer (n = 40), breast cancer (n = 27), esophageal cancer (n = 25), gastric cancer (n = 24), and brain tumors (n = 22). Overall, the cohort exhibited a typical real-world “pan-cancer + long-tail” distribution. Figure 2 summarizes the cancer type distribution of the final cohort.

### 3.2. Endpoint Overview

**Figure 3:**
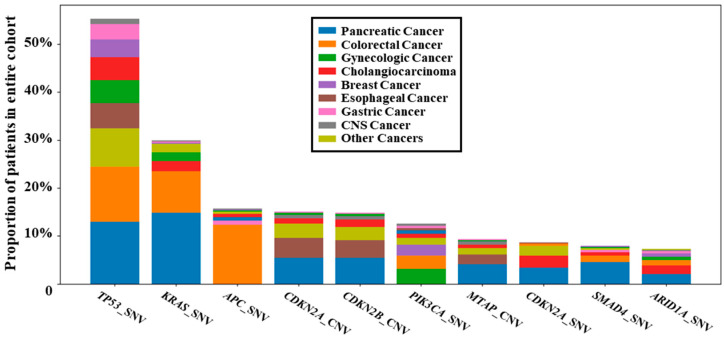
Prevalence of the top 10 genomic alterations in the pan-cancer clinical cohort. Stacked bars show the proportion of patients positive for each endpoint for the 10 most frequent curated binary alteration endpoints.

We curated approximately 1023 binary endpoints. High-frequency endpoints were enriched in canonical driver and tumor suppressor events, including *TP53*_SNV, *KRAS*_SNV, *APC*_SNV, *CDKN2A*_CNV, *CDKN2B*_CNV, *PIK3CA*_SNV, *MTAP*_CNV, *CDKN2A*_SNV, *SMAD4*_SNV, and *ARID1A*_SNV. The endpoint distribution was highly heterogeneous and long-tailed: beyond the third most frequent endpoint, prevalence dropped below 20%, indicating that severe class imbalance is the norm in real-world multi-endpoint modeling. When stratified by tumor type, endpoints showed distinct tumor-type contributions: *TP53*_SNV was broadly distributed across cancers; *KRAS*_SNV was enriched in pancreatic and colorectal cancers; *APC*_SNV was predominantly contributed by colorectal cancer; *PIK3CA*_SNV was enriched in gynecologic and breast cancers; and *CDKN2A/B*_CNV and *MTAP*_CNV were more prominent in pancreatic, biliary, and selected upper gastrointestinal tumors. The prevalence of the top 10 alteration endpoints stratified by tumor type is shown in Figure 3.

### 3.3. Foundation-Model Benchmarking

**Figure 4:**
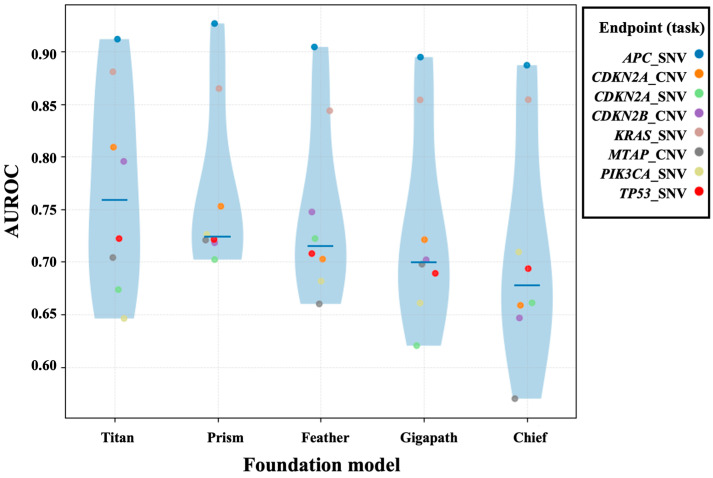
AUROC distributions of slide-embedding models across pathology foundation models. Violin plots summarize the endpoint-wise AUROC distributions obtained from a unified downstream MLP classifier trained on slide-level embeddings extracted by five pathology foundation models (Prism, Feather, Titan, Chief, and GigaPath). Colored points denote AUROCs for individual endpoints.

Without fine-tuning the foundation models, we benchmarked slide embeddings from five models using a unified downstream MLP evaluation, summarizing endpoint-wise AUROC distributions with scatter and violin plots. Overall, Titan achieved the highest median AUROC (0.77), suggesting more stable transferability in this pan-cancer cohort. Other models exhibited varying degrees of dispersion, potentially reflecting differences in pretraining data scale, aggregation strategies, and representational biases. Figure 4 compares endpoint-wise AUROC distributions across foundation models.

### 3.4. Patch-Level CLAM

**Figure 5:**
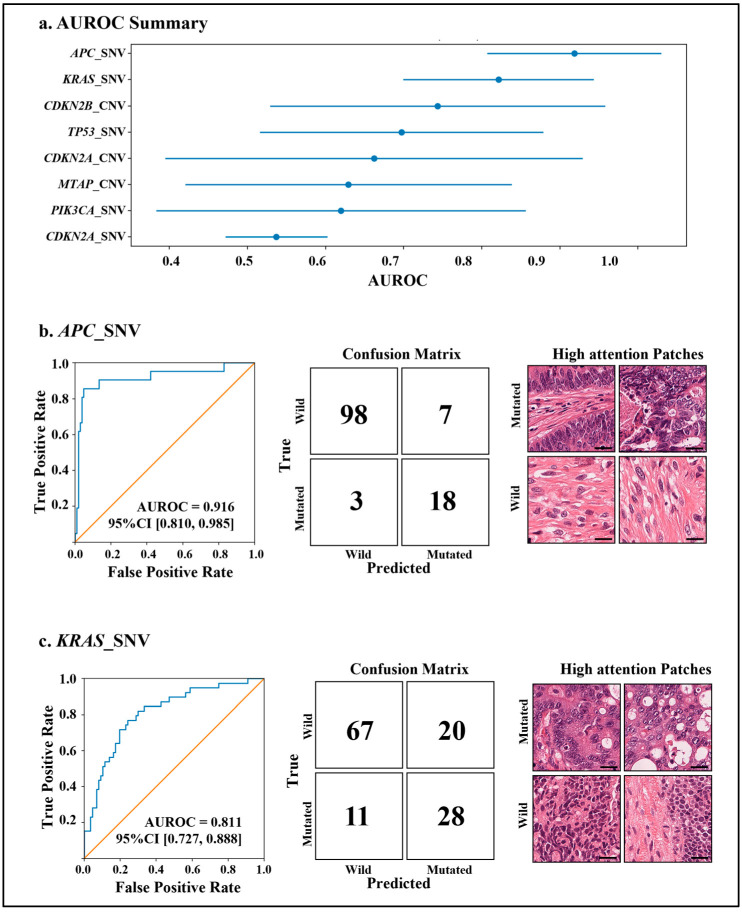
Performance of patch-feature-based CLAM models for genomic alteration prediction. (**a**) Overview of model discrimination across eight genomic alteration tasks, reported as AUROC with 95% confidence intervals on the held-out test set. (**b**,**c**) Representative tasks are shown for (**b**) APC_SNV and (**c**) KRAS_SNV, including AUROC curves (left) and confusion matrices (right) evaluated at the pre-specified operating threshold defined in the Methods, together with representative high-attention H&E patches for positive and negative predictions. The positive class denotes mutated samples (1), and the negative class denotes wild-type (0). Scale bars, 50 µm. AUROC, area under the receiver operating characteristic curve; CI, confidence interval; CLAM, clustering-constrained attention multiple-instance learning.

Using the best-performing patch feature system, we trained CLAM and reported AUROCs for key endpoints. *APC*_SNV achieved a pooled AUROC of 0.916, and *KRAS*_SNV achieved a pooled AUROC of 0.811. As an exploratory analysis, we noted that 78.4% of *APC*_SNV cases were colorectal cancer. Restricting to colorectal cases (n = 21) in the test set, the CLAM model achieved an AUROC of 0.714 (95% CI: 0.397–1.000). As a basic quantitative assessment, we reviewed the top 10 highest-attention patches for each correctly classified *APC*_SNV case within the colorectal cancer subset in the test set. In correctly predicted positive cases (n = 14), the mean proportion of tumor patches was 96.4%, compared with 70.0% in correctly predicted negative cases (n = 4), indicating that the model preferentially attends to tumor regions when predicting the presence of *APC* mutations. In addition, representative high-attention patches are visualized for correctly classified positive and negative cases. Figure 5 summarizes the test-set performance of patch-level CLAM across key endpoints.

## 4. Discussion

In this real-world pan-cancer cohort (437 cases across 26 tumor types) with long-tailed gene-panel endpoints, we show that without any fine-tuning, multiple pathology foundation models already provide slide embeddings that are “good enough” for genomic alteration prediction when paired with a lightweight MLP head. These foundation models have learned broadly transferable morphologic representations that encode mutation-associated tissue patterns, even under heterogeneous clinical conditions. Interestingly, the gap between “simple” and “heavy” models was not always large: in our benchmarking, a plain MLP on slide embeddings could reach MIL-level performance. Nevertheless, patch-level information is still relevant, especially for interpretability, since patch-level MIL can yield incremental gains for key endpoints and highlight discriminative regions consistent with morphologic assessment. The attention visualizations in Figure 5 provide additional evidence about where the model “looks”. For *APC*_SNV and *KRAS*_SNV, high-attention patches in mutated cases were predominantly localized to tumor regions, whereas in wild-type cases, high-attention often appeared in non-tumor compartments such as fibrotic stroma and lymphocyte-rich areas. This pattern suggests that, beyond the tumor, microenvironmental contexts (e.g., fibrosis and immune reaction) may also correlate with mutation status, reflecting shared biological programs or tumor–stroma/immune interactions. A prior multicenter deep-learning study [20] on colorectal cancer reported that *APC* mutation was primarily associated with attention to gland-forming adenocarcinoma, whereas *KRAS* mutation was associated with attention to villous adenomas with high-grade dysplasia and invasive adenocarcinoma; in contrast, low *KRAS* scores were often linked to TIL-rich regions, which is consistent with our observations.

This study also exposes the hard truths of real clinical data. Multi-endpoint modeling in gene-panel cohorts is inherently sparse and long-tailed, so performance for rare events can be unstable and overly sensitive to cohort composition, threshold choice, and split strategy. A second, more subtle risk is tumor-type confounding: in mixed tumor cohorts, models may first infer tumor type and then “borrow” prevalence priors to predict common alterations, creating shortcut learning that looks like generalization but may not survive external deployment. In our cohort, for instance, the *APC*_SNV pan-cancer AUROC of 0.916 dropped to 0.714 when evaluation was restricted to colorectal cancer alone, confirming that tumor-type confounding inflates overall performance. Looking forward, we think the most useful contribution is not “the best single model”, but a deployment-oriented workflow that matches how hospitals actually operate: slide embeddings for rapid, low-cost screening and representation selection, followed by patch-level study for interpretation where deeper learning and interpretability are clinically worthwhile. This two-stage paradigm is especially suited to settings with limited local sample sizes but many endpoints: you first benchmark foundation models cheaply to identify the most robust representation, and then concentrate compute and annotation effort on a small set of high-value tasks. The next step is to push this framework from retrospective benchmarking toward real clinical utility—prospective evaluation, calibration for decision support, and standardized interpretability readouts that pathologists can audit. As pathology models continue to improve, their most realistic role will be to triage and prioritize gene molecular testing in a way that is fast, scalable, and accountable.

While computational pathology has demonstrated remarkable progress in extracting molecular information from routine histology, it is important to emphasize that molecular sequencing retains its status as the gold standard for genomic alteration detection, and that regulatory-approved companion diagnostics guiding targeted therapy selection are built on validated sequencing-based assays that image-based predictions cannot substitute for in therapeutic decision-making. Several fundamental sources of uncertainty, such as tumor heterogeneity, slide quality, and cohort bias, limit the reliability of image-based deep learning genomic prediction. Furthermore, this study is limited by its monocentric design, which introduces potential batch effects, restricts sample size, and limits generalizability. Large-scale external validation across multiple institutions is therefore an important priority for future work. A recent study [21] systematically evaluating the genomic prediction performance of multiple pathology foundation models demonstrated that a substantial proportion of apparently high-performing models were capturing tumor type, histological subtype, or staging-related features as a proxy for mutation status. These limitations underscore that, even under favorable validation conditions, currently, the appropriate clinical role of such models is as a triage and decision-support tool to prioritize patients for confirmatory molecular testing, to provide a preliminary probabilistic estimate when sequencing results are pending or unavailable.

## 5. Conclusions

We present a five-year synthesis of computational pathology for genomic prediction and a real-world pan-cancer validation on 437 clinical cases, showing that pathology foundation models can deliver strong baseline genomic prediction performance without fine-tuning. Among five models benchmarked, Titan achieved the highest slide-level transfer stability, and patch-level CLAM yielded further gains for key endpoints. We propose a two-stage workflow, rapid slide-embedding screening followed by patch-level training. However, the monocentric design and tumor-type confounding limit generalizability, and prospective multi-institutional validation is needed before clinical adoption. This study offers a practical, scalable path toward real-world deployment of computational pathology for genomics-informed care.

## Figures and Tables

**Table 1:** Summary of pathology foundation models benchmarked in this study.

Model	Architecture (Slide Encoder)	Pretraining Data	Resolution
Feather	Attention-Based MIL	24,000 WSIs	512 × 512 px 20×
Gigapath	LongNet	171,189 WSIs	256 × 256 px 20×
Prism	Perceiver	587,196 WSIs	224 × 224 px 20×
Chief	Weakly Supervised Transformer	60,530 WSIs	256 × 256 px 10×
Titan	ViT	335,645 WSIs	512 × 512 px 20×

Abbreviations: MIL, Multiple Instance Learning; ViT, Vision Transformer; WSIs, Whole-Slide Images; px, pixels.

## Data Availability

The Trident framework is available at https://github.com/mahmoodlab/TRIDENT (accessed on 11 February 2026). The data that support the findings of this study are not publicly available due to privacy and ethical restrictions.
